# Pelvic Drop Changes due to Proximal Muscle Strengthening Depend on Foot-Ankle Varus Alignment

**DOI:** 10.1155/2019/2018059

**Published:** 2019-05-12

**Authors:** Aline de Castro Cruz, Sérgio Teixeira Fonseca, Vanessa Lara Araújo, Diego da Silva Carvalho, Leonardo Drumond Barsante, Valéria Andrade Pinto, Thales Rezende Souza

**Affiliations:** ^1^Graduate Program in Rehabilitation Sciences, Universidade Federal de Minas Gerais, Belo Horizonte, Minas Gerais, Brazil; ^2^Department of Physical Therapy, Universidade Federal de Minas Gerais, Belo Horizonte, Minas Gerais, Brazil

## Abstract

**Background:**

Strengthening of hip and trunk muscles can modify pelvis and hip movements. However, the varus alignment of the foot-ankle complex (FAC) may influence the effects of muscle strengthening, due to the relationship of FAC alignment with pelvic and hip kinematics. This study evaluated the effects of hip and trunk muscle strengthening on pelvis and hip kinematics during walking, in subgroups with larger and smaller values of FAC varus alignment. In addition, this study evaluated the effects of hip and trunk muscle strengthening on hip passive and active properties, in the same subgroups.

**Methods:**

Fifty-three women, who were divided into intervention and control groups, participated in this nonrandomized controlled trial. Each group was split into two subgroups with larger and smaller values of FAC varus alignment. Hip and trunk muscle strengthening was performed three times a week for two months, with a load of 70% to 80% of one repetition maximum. Before and after strengthening, we evaluated (1) pelvis and hip excursions in the frontal and transverse planes during walking, (2) isokinetic hip passive external rotator torque, and (3) isokinetic concentric and eccentric peak torques of the hip external rotator muscles. Mixed analyses of variance (ANOVAs) were carried out for each dependent variable related to walking kinematics and isokinetic measurements (*α* = 0.05).

**Results:**

The subgroup with smaller varus alignment, of the intervention group, presented a reduction in pelvic drop after strengthening (*P* = 0.03). The subgroup with larger varus alignment increased pelvic drop after strengthening, with a marginal significance (*P* = 0.06). The other kinematic excursions did not change (pelvic anterior rotation *P* = 0.30, hip internal rotation *P* = 0.54, and hip adduction *P* = 0.43). The intervention group showed increases in passive torque (*P* = 0.002), peak concentric torque (*P* < 0.001), and peak eccentric torque (*P* < 0.001), independently of FAC alignment. These results suggest that FAC varus alignment influences the effects of strengthening and should be considered when hip and trunk muscle strengthening is used to reduce pelvic drop during walking.

## 1. Introduction

Strengthening of hip and trunk muscles has been used to modify pelvis and hip excessive movements [1–4] since they may be involved in the production of injuries of the hip [5, 6] and the lumbopelvic complex [7]. Pelvic drop, axial rotation, hip adduction, and hip internal rotation occur in the first half of the stance phase of walking [8, 9]. Muscle strengthening may be used in an attempt to reduce amplitude of these motions. For example, strengthening of the hip external rotators and abductors may increase passive and active (eccentric) mechanical resistance against hip internal rotation and adduction [3, 4, 10]. In addition, strengthening of trunk rotators and lateral flexors may increase passive and eccentric resistance against pelvic drop and axial rotation [3, 4, 8, 9]. Gains in concentric strength of these muscles could also facilitate the production of hip external rotation and abduction, as well as pelvic raise. These movements take place subsequently in the second half of the stance phase [9]. Despite these theoretical benefits, previous studies have demonstrated no effects of hip strengthening on the kinematics [11] and kinetics [12] of the pelvis and hip during walking.

Biomechanical characteristics other than the active and passive functions of the hip and trunk muscles could influence lower limb kinematics in weight-bearing tasks [10, 13] and affect the kinematic changes produced by the strengthening. Varus alignment of the foot-ankle complex (FAC), measured in non-weight-bearing, constitutes a biomechanical factor that influences the magnitude of FAC pronation during walking [14, 15]. By its turn, the magnitude of FAC pronation during weight-bearing tasks may influence hip and pelvis kinematics [16–20]. Higher FAC pronation values are related to higher magnitudes of hip internal rotation and adduction [17–20] and pelvic drop [16, 19]. Therefore, the magnitude of FAC varus alignment (and the magnitude of FAC pronation) may be an individual biomechanical characteristic that influences the effects of muscle strengthening on changing pelvis and hip motions. For example, larger values of FAC varus alignment may result in greater FAC pronation [14, 15] and produce greater hip adduction and pelvic drop [16–20] during walking. Thus, the presence of larger varus could make it difficult to produce an amplitude reduction of these motions, after proximal muscle strengthening.

The objective of this study was to investigate the influence of FAC varus alignment on possible changes resulting from muscle strengthening at the hip (mainly abductors and lateral rotators) and trunk (mainly abdominals and latissimus dorsi). We considered possible changes in (1) the motion excursions of the pelvis and hip in the frontal and transverse planes, during gait, and (2) hip muscle strength and passive torque. The main study hypotheses were that a subgroup with larger FAC varus alignment would have smaller or no reductions in the excursions of pelvis and hip motions. Changes in hip strength and passive torque were also investigated to help understand possible kinematic effects. A secondary study objective was to verify if individuals with larger FAC varus alignment have larger foot pronation during walking, as previously observed [14, 15].

## 2. Methods

### 2.1. Participants

Fifty-three women, who were divided into intervention and control groups, participated in this nonrandomized controlled trial. Both groups were divided into subgroups of larger and smaller varus magnitudes. The participants were selected by means of convenience sampling. The number of participants was calculated based on an expected moderate effect size (*f* = 0.3), with a level of significance of 0.05 and a desired statistical power of 0.80. According to this analysis, approximately 58 individuals would be needed. This study was approved by the institution's Research Ethics Committee (CAAE–0427.0.203.000-11).

The inclusion criteria were (1) female, since women present greater pelvic drop and hip adduction compared to men [19, 20] and may be more frequently subjected to interventions intended to reduce pelvic and hip motions; (2) age between 18 and 35 years, to avoid the impact of age on hypertrophy [21]; (3) body mass index (BMI) less than or equal to 25 kg/m^2^ to facilitate palpation of anatomical landmarks for the kinematic model creation; (4) absence of self-reported musculoskeletal symptoms or injuries in the last three months, to prevent the impact of pain and previous injury on the assessed movement pattern; (5) no practice of physical exercise in the last three months, to remove possible confounding effects of other physical exercises on the strengthening protocol used in this study; and (6) presence of normal range of motion of hip internal rotation (from 34° to 71°) and hip external rotation (from 25° to 56°) [22]. People with hip internal and external rotation restrictions could have difficulties to perform the strengthening exercises properly. In addition, these individuals might also have anatomical abnormalities such as anteversion or retroversion of the femoral neck, which could influence changes in hip kinematics after muscular strengthening. The exclusion criteria were (1) adherence lower than 80% of the training sessions of the strengthening program. The time needed for hypertrophy gains in the lower limbs of women is about six weeks, after the beginning of training, which corresponds to 80% of the duration of the training protocol used in our study [23]; (2) engagement in physical exercise during the study period, to prevent possible confounding effects of other physical exercises on the strengthening protocol used in this study (physical exercise was considered as the practice of activities or training aiming to improve physical fitness or health); (3) inability to keep the hip muscles relaxed during the passive torque test; and (4) presence of pain during the assessments and inability to perform the tests correctly.

FAC varus alignment measurements were performed in both lower limbs of the participant. Allocation in the control and intervention groups was performed according to the availability of participants to perform the intervention. For allocation in the larger and smaller FAC subgroups, the control and intervention groups were divided in relation to varus values by means of the 50th percentile. However, in order to guarantee that the varus alignment values were similar between the control and intervention groups, it was necessary to match the groups according to varus values. Thus, the participants from the intervention group were evaluated, and the definition of which limb should be analyzed was random (drawing). For the control group, the varus alignment was evaluated in both limbs, and the limb analyzed was selected to match the varus value of a participant from the intervention group. This matching procedure was done to guarantee that the subgroups from the intervention and control groups were similar according to the magnitude of FAC varus.

**Figure 1:**
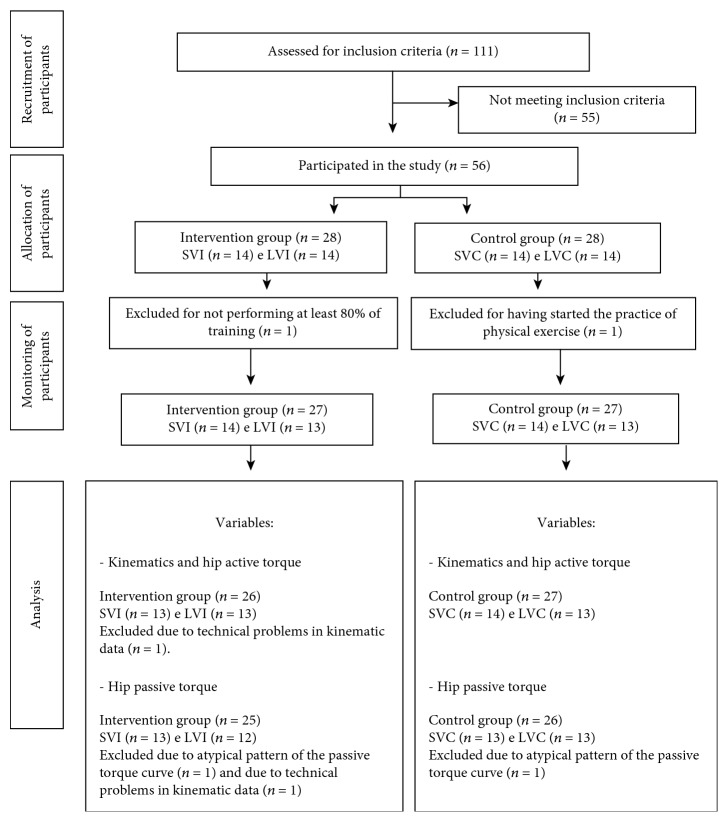
Flow diagram showing the number of participants at each stage of the study. LVI: larger varus in the intervention group; SVI: smaller varus in the intervention group; LVC: larger varus in the control group; SVC: smaller varus in the control group.

Initially, fifty-six women were included in the study. The participants signed an informed consent form to participate in the study. The intervention group (*n* = 26) was divided into two subgroups: one with smaller varus alignment (*n* = 13) and another with larger varus alignment (*n* = 13). The control group (*n* = 27) was also split into subgroups with smaller varus alignment (*n* = 14) and larger varus alignment (*n* = 13) (Figure 1). The groups were divided according to the median varus alignment values to equally separate them into subgroups of larger and smaller varus alignments. In addition, this division also allowed to investigate whether the varus values of the subgroups are associated with FAC pronation amplitude during gait.

Independent *t*-tests showed that FAC alignment values were significantly different between the subgroups of the intervention group (*P* < 0.001) and between the subgroups of the control group (*P* < 0.001). The smaller varus subgroups, in the control and intervention groups, did not show significant differences in varus alignment (*P* = 0.57). The larger varus subgroups, in the control and intervention groups, did not show significant differences in varus alignment neither (*P* = 0.71). The characteristics of the subgroups are indicated in Table 1.

### 2.2. Evaluation of Foot-Ankle Complex (FAC) Alignment: Forefoot-Shank Angle

The FAC alignment was assessed by means of a clinical measure that combines the varus/valgus alignment of the FAC (forefoot, rearfoot, and shank alignments) and midfoot inversion mobility [15, 24]. This clinical measure provides the forefoot-shank angle, measured in open chain (see Figure S1 in Supplementary Materials). In the present study, for simplification, we considered larger values of the forefoot-shank angle as larger values of FAC varus alignment. The mean of three forefoot-shank angle measures was calculated and used for analysis. The intraexaminer reliability of this measure was evaluated with ten individuals who performed two evaluations with a one-week interval, and the intraclass correlation coefficient (ICC) obtained was 0.93.

### 2.3. Evaluation of Walking Kinematics

A three-dimensional motion analysis system Codamotion (Charnwood Dynamics, Rothley, England) was used. Motion of the pelvis, thigh, shank, rearfoot, and forefoot was captured with clusters of active tracking markers that were placed on each of these body segments [4]. Moreover, anatomical markers (two proximal and two distal markers) were used in each segment for the kinematic model definition [4, 25]. Initially, the participant remained in orthostatic position for a static data collection of five seconds. The position of the participant's feet was drawn on a paper to be reproduced during reassessment. This static posture data with both tracking and anatomical markers was later used to create the kinematic model. After the static data collection, the participant walked on an electric treadmill (ProAction G635 Explorer, BH Fitness; Vitoria-Gasteiz, Alava, Spain) with the tracking markers only. Thirty consecutive walking cycles were collected at a sampling rate of 100 Hz. The participant was asked to walk at her self-selected comfortable speed, in the first evaluation. The same speed was used in the reevaluation (i.e., after the intervention period). The mean and range of speed of the subgroups were as follows: smaller varus intervention group (3.08 km/h (SD 0.73), 2.00 to 4.00), larger varus intervention group (3.15 km/h (SD 0.59), 2.00 to 4.00), smaller varus control group (3.07 km/h (SD 0.55), 2.00 to 3.50), and larger varus control group (2.73 km/h (SD 0.52), 2.00 to 3.50). An initial statistical analysis was done to verify if the velocities were different among the subgroups. A two-way ANOVA was performed with the factors group (control and intervention) and varus alignment (smaller and larger). Since the velocity was exactly the same in the evaluation and reevaluation, the condition was not a factor for this analysis. The interaction group × varus alignment revealed that the velocity was not different among the subgroups (*P* = 0.214).

Visual 3D software (C-Motion Inc., Rockville, Maryland, USA) was used to process the kinematic data. The data were filtered with a fourth-order, Butterworth, zero-lag, low-pass filter, with a cut-off frequency of 6 Hz. From the static posture data, a rigid body kinematic model of six degrees of freedom was created [25–27] and applied to the walking trial. The pelvis angle (pelvic motion in relation to the global coordinate system) and hip angle (thigh motion in relation to the pelvis) were calculated in the frontal and transverse planes during the stance of walking. Furthermore, rearfoot-shank and forefoot-shank angles (rearfoot and forefoot motions in relation to the shank) were calculated in the frontal plane during the stance of walking. These angles were created with the following Cardan sequence: lateral-medial, anterior-posterior, and superior-inferior [25].

The stance phase of walking was defined as the period from the instant the calcaneus contacted the ground until the instant the toes left the ground. These events were established by two examiners who concurrently observed the anteroposterior displacement curves of rearfoot and forefoot tracking markers [28]. Ten to sixteen walking stance phases without any marker tracking loss were analyzed for each participant. This intersubject variation in the number of analyzed trials was due to an uneven number of marker losses among the subjects. However, none of the subjects had less than 10 trials included in the analysis [29].

Motion excursions during the stance phase of walking were calculated for pelvic anterior rotation (transverse plane), pelvic drop (frontal plane), hip internal rotation (transverse plane), and hip adduction (frontal plane). Motion excursion was computed as the difference between the angle obtained in the first frame of stance (i.e., at initial contact) and the peak angle within the stance phase. For statistical analyses, the outcome variables were the average excursions obtained from the included trials of each participant. Rearfoot-shank and forefoot-shank eversion excursions (frontal plane) were calculated only for the prestrengthening evaluation, since these variables were used only to verify if individuals with larger varus alignment show greater FAC pronation during walking.

The test-retest reliability of all outcome variables (i.e., excursions of the pelvis, hip, rearfoot-shank, and forefoot-shank motions) was evaluated in a pilot study with ten participants in two evaluations with a one-week interval. This analysis showed moderate to good reliability (intraclass correlation coefficients ranging from 0.77 to 0.89).

### 2.4. Isokinetic Evaluation: Passive Torque and Hip Muscle Strength

Hip passive torque and muscle strength were measured with an isokinetic dynamometer Biodex 3 Pro (Biodex Medical Systems, Shirley, USA) at a sampling rate of 100 Hz. During passive hip measurement, the dynamometer was in the passive mode, and a surface electromyography system (ME6000, Mega Electronics Inc., Kuopio, Finland) was used to ensure that hip muscles were relaxed. Electromyographic data were collected at a sampling rate of 1000 Hz and recorded using the MegaWin 3.0 software (Mega Electronics Inc., Kuopio, Finland). Active surface electrodes were placed on the following muscles: gluteus maximus, gluteus medius, biceps femoris, tensor fascia lata, and adductor magnus [30].

For the measurement of the hip passive torque during internal rotation, the participant was positioned in prone, with the knee flexed at 90° and the tibial tuberosity aligned with the axis of rotation of the isokinetic dynamometer. The upper limbs of the participant were placed next to the trunk, and a belt was used to stabilize the pelvis. The equipment's attachment moved the participant's hip from 25° of external rotation to 25° of internal rotation, at an angular velocity of 5°/s [4]. The examiner instructed the participant to remain relaxed and not to resist and/or assist hip motion during the test. Before the test, five repetitions of the movement were made for tissue viscoelastic accommodation and participant familiarization. Moreover, electromyographic signals of the muscles were recorded with the participant resting in static position. During the test, three valid measures of hip passive torque were performed. At each repetition of the test, the electromyographic data were extracted and processed in MATLAB software (The MathWorks Inc.). The data was filtered using a fourth-order, Butterworth, bandpass filter, with cut-off frequencies of 10 and 500 Hz. The signal collected during the participant's rest was compared to the signal obtained during each test. The repetitions with muscular activity were defined as those in which the electromyography signal was equal to or greater than the mean plus two standard deviations of the signal captured at rest. When muscle contraction occurred, a new repetition of the test was performed.

The evaluation of the active maximum concentric and eccentric hip external rotation torques was performed with the participant in the same position of the passive torque test, except for the upper limbs. The participant was instructed to hold a belt placed under the chair and keep the shoulders and elbows flexed to stabilize the trunk during the test. For familiarization, before the test, the participant performed the movement with submaximal force for five repetitions. The tests were performed from 30° of internal rotation to 20° of external rotation at an angular velocity of 30°/s [4]. During external rotation, the hip muscles contracted concentrically. During internal rotation, the muscles contracted eccentrically. The participant received instructions and verbal encouragement to produce maximum strength. Three sets of five repetitions were performed, and the concentric and eccentric torques of each repetition were recorded.

For data reduction, the participant's shank and foot lengths were measured. In addition, a repetition of the test without the participant was performed to record the torque produced by the weight of the equipment's lever arm.

The data obtained by the isokinetic dynamometer were processed with a routine developed in MATLAB. The signals were filtered with a fourth-order, Butterworth, low-pass filter, with a cut-off frequency of 1.25 Hz. The torques generated by the shank, foot, and dynamometer's lever arm weights were subtracted from the total torque [4].

For the passive torque, the mean torque produced during the first 20° of hip internal rotation was calculated, in Newton meters (Nm), for each test repetition [4]. This amplitude of hip internal rotation was chosen as an approximation of the average range of hip rotation during walking [31]. For the active torques, the peak values of the concentric and eccentric torques were calculated for each repetition. For statistical analysis, the mean of the three repetitions was used, for the passive, concentric, and eccentric torques.

### 2.5. Muscle Strengthening Protocol

Hip and trunk muscle strengthening was performed three times a week for two months. The period between the prestrengthening evaluation and the poststrengthening evaluation was two months, with a maximum limit of two months and one week. The training days were chosen according to the participant's availability. The load for the exercises was set at 70% to 80% of one repetition maximum (1RM) [32], as this load level is recommended when hypertrophy is aimed [32]. During the 1RM test, the examiner observed the movement to ensure that the participant performed the exercise throughout the full range of motion without compensatory movements (i.e., movement components performed by muscles other than the muscles being tested). For each exercise of the protocol, three sets of eight to nine repetitions were performed at moderate velocity (about 3 seconds for the isotonic cycle) with a one-minute rest between sets [32].

**Figure 2:**
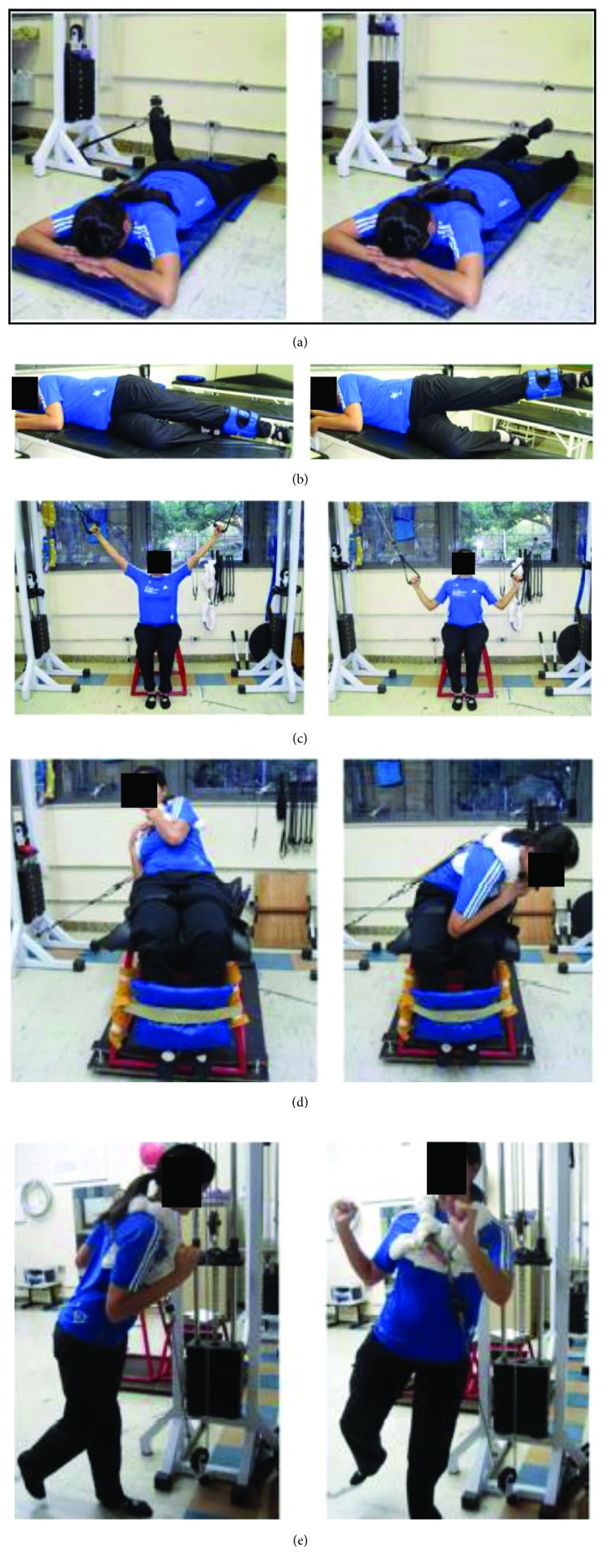
Strengthening exercises of the hip and trunk muscles: (a) hip external rotators, (b) gluteus medius, (c) latissimus dorsi, (d) abdominal oblique and quadratus lumborum, and (e) hip and trunk rotators and extensors in closed kinematic chain. Source: [4].

The load was increased by 5 or 10% when the participant was able to perform three sets of nine repetitions for two consecutive sessions [32]. In the fifth week, the eccentric training of hip external rotators and abductors was increased to 90% or 100% of 1 concentric RM [33, 34]. Bilateral isotonic strengthening was performed in full range of motion for the following muscles: (a) hip external rotators, (b) gluteus medius, (c) latissimus dorsi, (d) oblique and rectus abdominis and quadratus lumborum, and (e) hip and trunk rotators and extensors in closed kinematic chain [4]. It was expected that the strengthening of these muscles could reduce excessive motion excursions of the pelvis and hip in the frontal and transverse planes. Hip external rotators and abductor muscles could resist hip excessive internal rotation and adduction [8, 9]. The trunk muscles also have the potential to resist hip adduction and internal rotation [8, 9]. Tension in the latissimus dorsi muscle can be transmitted to the gluteus maximus through the thoracolumbar fascia and increases hip resistance against internal rotation [35]. The oblique abdominal muscles can reduce ipsilateral pelvic drop and contralateral hip adduction [8, 9]. Therefore, the open-chain exercises depicted in Figure 2 were chosen to selectively strengthen the desired muscles.

The last exercise (Figure 2(e)) was initiated only in the third week with minimal load (5 kg), aiming to learn the correct execution of the movement. In the fifth week, the load of this exercise was increased to 70% or 80% of 1RM [4]. The purpose of this exercise was to promote a more global strengthening, for the muscles that can help extend and rotate the hip and trunk. It was performed in a weight-bearing situation such as the stance phase of walking. Although this last exercise was performed in weight bearing, one objective of the present study was to investigate the kinematic effects of changes in the active and passive functions of the muscles regardless of the exercises being performed in open- or closed-chain situations.

### 2.6. Statistical Analysis

Mixed analyses of variance (ANOVAs) were carried out for each dependent variable: excursion of pelvic anterior rotation, pelvic drop excursion, hip internal rotation excursion, and hip adduction excursion; passive hip torque; and concentric and eccentric hip external rotator torques. Each ANOVA had two between-subject effects with two levels (varus alignment: larger and smaller; group: control and intervention) and one within-subject effect with two levels (condition: pre- and postintervention). Each mixed ANOVA performed for each dependent variable generated the following interactions of interest: group × condition and group × condition × varus alignment. When a significant difference was found in the interaction effect, preplanned contrasts were used to identify which group and subgroup comparisons showed significant differences.

After verifying data normality by means of Shapiro-Wilk tests, independent *t*-tests were performed to compare the rearfoot-shank eversion angle (data with normal distribution) between subgroups of smaller and larger varus alignments. Moreover, the Mann-Whitney test was carried out to compare the forefoot-shank eversion angle (data with nonnormal distribution) between subgroups of smaller and larger varus alignments. For all analyses, a type 1 error probability of 5% (*α* = 0.05) was considered.

## 3. Results

Descriptive data of all outcome variables are presented in Supplementary Materials (see Table S1).

### 3.1. Pelvis and Hip Kinematics

ANOVAs revealed no significant group × condition interaction effects for the excursions of pelvic anterior rotation (*P* = 0.63), pelvic drop (*P* = 0.95), hip adduction (*P* = 0.85), and hip internal rotation (*P* = 0.48) during walking (Table 2). Therefore, the intervention and control groups did not show changes after the intervention period.

However, a significant effect for the interaction group × condition × varus alignment was demonstrated for pelvic drop (*P* = 0.01). The contrasts showed that the subgroup of smaller varus alignment reduced pelvic drop after strengthening (*P* = 0.03) (Table 3). For the subgroup of larger FAC varus, a marginal *P* value (*P* = 0.06) was observed, which shows a tendency of increase in pelvic drop during walking (Table 3). In addition, both control groups with smaller and larger varus alignments did not show significant differences in pelvic drop after eight weeks (*P* = 0.70 and *P* = 0.81, respectively). Thus, the intervention group showed a change after the intervention period, for pelvic drop, only when it was divided into smaller and larger varus alignment subgroups. And the same subgroups in the control group did not show any changes.

### 3.2. Isokinetic Variables

For the passive hip torque, ANOVAs demonstrated significant effects for group × condition interaction (*P* = 0.002) and no significant effect for group × condition × varus alignment interaction (*P* = 0.98) (Table 4). The contrasts revealed that the intervention group increased the passive torque (*P* = 0.001) after muscle strengthening, and the control group did not change after eight weeks (*P* = 0.25) (Table 5). Therefore, the intervention group showed a change while the control group did not, and these results were not dependent on the varus alignment.

ANOVAs showed significant group × condition interactions for the concentric (*P* < 0.001) and eccentric (*P* < 0.001) peak torques of the hip external rotators (Table 4). However, no significant effect was found for the interaction group × condition × varus alignment (concentric torque *P* = 0.21 and eccentric torque *P* = 0.10) (Table 4). The contrasts revealed that the intervention group increased the concentric peak torque (*P* < 0.001) and the eccentric peak torque (*P* < 0.001) after the strengthening program (Table 5). No change in concentric (*P* = 0.62) and eccentric (*P* = 0.22) torques was observed in the control group after eight weeks (Table 5). Thus, the intervention group showed changes while the control group did not, and these results were not dependent on the varus alignment.

### 3.3. Foot-Ankle Complex (FAC) Kinematics

The Mann-Whitney test revealed that, in the preintervention condition, participants with smaller varus alignment had lower excursions of forefoot-shank eversion (9.86 ± 4.09) compared to the participants with larger varus alignment (14.88 ± 4.17) (*P* < 0.001). Moreover, the independent *t*-test showed no significant difference in rearfoot-shank eversion during stance phase of walking between the groups with larger and smaller varuses in the preintervention condition (9.18 ± 2.78 and 7.80 ± 3.25, respectively) (*P* = 0.10). Therefore, the participants with smaller varus alignment showed lower forefoot-shank eversion than the participants with larger varus alignment.

## 4. Discussion

Hip and trunk muscular strengthening significantly reduced the excursions of pelvic drop during walking, exclusively in the subgroup of individuals with smaller values of FAC varus alignment. There was also an increase in pelvic drop, with a tendency towards statistical significance, in the subgroup with larger FAC varus alignment. When considering all individuals (without subgrouping by varus alignment), no kinematic changes were found in the intervention group or in the control group, after the intervention period. Muscular strengthening increased hip active torques (concentric and eccentric) and hip passive torque, independently of FAC alignment. Consistent with previous studies [14, 15], the assumption that women with larger FAC varus alignment would have greater excursions of FAC pronation was confirmed for the forefoot-shank eversion excursion. The present findings showed that the effect of the muscle strengthening program on pelvic drop depended on the magnitude of the FAC varus alignment. The following were observed: (a) reduction in pelvic drop only in the subgroup of women with smaller varus alignment and (b) a tendency for pelvic drop increase in the subgroup of women with larger varus alignment.

Previous studies that investigated consequences of hip strengthening on the kinematics of the pelvis [11] and hip kinetics [12] during walking found no effects. Kendall et al. [11] identified that strength increases of hip abductors did not reduce the pelvic drop during walking, in patients with nonspecific low back pain. This finding coincides with the results of the present study, when all participants were considered together (Table 2). This is a possible consistency across findings, although there are important methodological and sample differences among the studies. In contrast to the observed absence of kinematic effects of strengthening, the present study found significant effects on pelvic kinematics, when FAC varus alignment was considered.

Increases in torque did not differ between individuals with larger and smaller varus alignments. Thus, the pelvic drop reduction observed only in the subgroup of individuals with smaller FAC varus alignment does not seem to be related with particular increases of active and passive hip torques in this subgroup. Thus, other factors may also contribute to the control of the pelvis motion in the frontal plane. It has been reported that individuals with larger varus alignment show greater FAC pronation [14, 15] and tend to have greater hip adduction and pelvic drop [19]. The relation between increased FAC varus alignment and increased forefoot eversion during walking was confirmed in the present study. Therefore, the present results suggest that torque increases after strengthening interacted with the presence of smaller FAC varus alignment to reduce pelvic drop. These findings suggest that the strengthening program is recommended to reduce pelvic drop in individuals with smaller FAC varus alignment.

The subgroup with larger FAC varus alignment had a tendency to increase pelvic drop after strengthening, with a marginal significance (*P* = 0.06). As the effect size was moderate and the achieved statistical power was low (less than 80%) (Table 3), it is possible that a larger sample could reach statistical significance. In addition, this subgroup demonstrated a high variability in pelvic drop changes, which may have also reduced the study's statistical power (Table 3). This possible increase in pelvic drop may be due to hip and trunk adaptations to factors not measured in this study, which could have happened in response to the stimuli provided by the muscle strength training. For example, hip abductor passive torque may be reduced when the muscles are used in greater lengths during daily activities [36]. People with increased foot pronation, such as those with larger varus alignment, have greater hip adduction [19, 20]. This along with the muscle training might have led to adaptations in the abductor muscles. However, this is very speculative and would need further investigation. Therefore, further studies are needed to better investigate the effects of hip and trunk strengthening in people with larger varus alignment. At this point, caution is advised in the use of the studied muscle strengthening program for individuals with larger FAC varus alignment, considering the possible increase in pelvic drop.

The increases in hip (external rotator) passive torque and active concentric and eccentric torques in the intervention group did not influence hip excursion in the frontal and transverse planes and pelvis excursion in the transverse plane. Perhaps, the implementation of neuromuscular training during walking and/or the use of weight-bearing exercises performed in conditions more similar to the stance phase of walking could produce more consistent results. These interventions would allow the motor system to explore the new resources provided by strengthening (i.e., greater passive torque and capability of producing greater active torques). Functional training in addition to muscle strengthening might be more effective in changing excursions of pelvic anterior rotation and hip internal rotation and adduction [2, 3, 37–39]. In addition, strengthening programs with greater training volume (i.e., capable of generating greater modifications in the musculoskeletal tissue and muscular properties) could be necessary to produce kinematic effects on the pelvis in the transverse plane and the hip in the frontal and transverse planes. Although the increase in maximum active torque produced during an isokinetic test does not necessarily mean that an active torque increase will occur during walking [40], the increase in passive joint torque measured isokinetically will contribute to the motions in walking. Hence, greater increases in passive torque could result in greater kinematic changes after strengthening. However, it is important to note that the strengthening program used in this study can be considered to have a medium-to-high volume training [32].

It should be noted that the average 0.56° reduction in pelvic drop observed in the smaller varus subgroup could be viewed as a small change with limited clinical relevance. However, this change represents approximately 13% of the average total pelvic drop excursion observed. Pelvic and hip kinematic changes of similar magnitudes may be relevant. For example, runners with patellofemoral pain have 11% more hip adduction compared to healthy controls [41]. Gait retraining reduced pelvic drop in 24% and reduced pain in runners with patellofemoral pain [42]. Since hip adduction is related to closed-chain pelvic drop [9], the reduction in pelvic drop found in the present study might contribute to clinical improvements. In addition, a similar rationale can be applied for the increase in pelvic drop observed in the larger varus subgroup (average increase of 0.56°). It is also possible that this small change could be potentially harmful [5, 6]. However, this needs to be subjected to further investigation.

Some limitations of the present study can be pointed out. First, there is a technical difficulty to adequately capture thigh kinematics, especially in the transverse plane, due to large errors related to soft tissue artifacts [25, 43]. In addition to its contribution to greater data variability, this difficulty may have prevented the detection of strengthening effects in hip kinematics. Unfortunately, this is a limitation of the noninvasive motion-tracking procedure that is currently recommended for hip kinematic assessment [43]. Another limitation was the nonrandom allocation of the participants in groups. Participants were allocated to groups according to their availability to participate in the program (convenience method), which was necessary to make the study data collection feasible. Finally, only able-bodied and asymptomatic women participated in the study, which limits results' generalization. However, the study results may apply to programs that are aimed at preventing orthopedic problems in the lumbopelvic complex, in this population. Future studies could evaluate the effect of hip and trunk muscle strengthening in patients with pelvis and hip dysfunctions/injuries during walking in both sexes to investigate whether men and individuals with dysfunctions/injuries have the same responses to the strengthening protocol.

The results of this study demonstrated that individual characteristics such as the FAC varus alignment influence kinematic effects of a proximal muscle strengthening program. Thus, the present findings point to the importance of considering individual characteristics when choosing an intervention that is aimed at changing movement patterns. FAC varus alignment assessment should be carried out when the purpose of a proximal muscle strengthening protocol is to reduce pelvic drop during walking.

## 5. Conclusion

Hip and trunk muscle strengthening reduced pelvic drop excursion only in women who have smaller FAC varus alignment, during walking. In addition, there was a tendency for pelvic drop increases in women who have larger FAC varus alignment. The strengthening increased passive and active hip torques in the transverse plane, regardless of the magnitude of FAC varus alignment. These results indicate that specific individual characteristics, such as FAC alignment, may influence the kinematic effects of proximal muscle strengthening. Thus, FAC alignment may influence the decision to use the studied hip and trunk muscle strengthening to change pelvic drop.

## Figures and Tables

**Table 1:** Characteristics of the subgroups.

Groups	Subgroups	Age (years)	BMI (kg/m^2^)	FAC varus (°)	Evaluated limb
		Mean (SD)	Mean (SD)	Mean (SD)	
Intervention	Smaller varus	21 (2.95)	20.80 (1.57)	9.51 (4.44)	Left (*n* = 7) Right (*n* = 6)
	Larger varus	23 (3.88)	21.39 (2.24)	22.08 (4.68)	Left (*n* = 7) Right (*n* = 6)
Control	Smaller varus	22 (1.73)	20.63 (2.10)	10.54 (4.75)	Left (*n* = 2) Right (*n* = 12)
	Larger varus	21 (1.97)	21.31 (2.10)	21.45 (3.79)	Left (*n* = 7) Right (*n* = 6)

SD: standard deviation; BMI: body mass index; FAC: foot-ankle complex.

**Table 2:** Significance of the kinematic variables for the interactions of interest.

Variables	Group × condition		Group × condition × varus alignment	
	*P*	*F*	*P*	*F*
Pelvis frontal	0.95	0.004	0.01^∗^	7.12
Pelvis transverse	0.63	0.23	0.30	1.11
Hip frontal	0.85	0.03	0.43	0.63
Hip transverse	0.48	0.50	0.54	0.38

^∗^ *P* ≤ 0.05; *F*: *F* value for the interaction in mixed ANOVA.

**Table 3:** Mean, SD, and significance for the pelvic drop excursions of the varus alignment subgroups, in the intervention and control groups, before and after strengthening.

Subgroups	Condition	Pelvic drop excursion (°) Mean (SD)	*P*	*t*	Effect size Cohen's *d*	Power
SVI	Preinterv.	5.62 (2.11)	0.03^∗^	2.42	0.68	0.38
	Postinterv.	5.06 (2.18)				
LVI	Preinterv.	6.24 (2.45)	0.06	-2.11	0.58	0.49
	Postinterv.	6.80 (2.17)				
SVC	Preinterv.	4.95 (1.42)	0.70	-0.39	0.10	0.07
	Postinterv.	5.04 (1.42)				
LVC	Preinterv.	3.25 (2.03)	0.81	0.25	0.07	0.06
	Postinterv.	3.20 (2.16)				

SD: standard deviation; *t*: *t* value for the *t*-test (contrast); interv.: intervention; (°): degrees; LVI: larger varus intervention; SVI: smaller varus intervention; LVC: larger varus control; SVC: smaller varus control. ^∗^ *P* ≤ 0.05 in the comparison between the pre- and postintervention conditions of the varus alignment subgroups, in the control and intervention groups.

**Table 4:** Significance of the interactions of interest for the passive and active torques.

Variables	Group × condition		Group × condition × varus alignment	
	*P*	*F*	*P*	*F*
Hip passive torque	0.002^∗^	10.96	0.98	0.001
Concentric torque peak	<0.001^∗^	28.01	0.21	1.64
Eccentric torque peak	<0.001^∗^	41.27	0.10	2.82

^∗^ *P* ≤ 0.05; *F*: *F* value for the interaction in mixed ANOVA.

**Table 5:** Mean, SD, and significance of mean hip passive torque and concentric and eccentric peak torques before and after strengthening, in the intervention and control groups.

Groups	Condition	Passive mean torque (Nm)	*P*	*t*	Concentric peak torque (Nm)	*P*	*t*	Eccentric peak torque (Nm)	*P*	*t*
		Mean (SD)			Mean (SD)			Mean (SD)		
Intervention	Preinterv.	1.28 (0.59)	0.001^∗^	-3.97	25.16 (5.03)	<0.001^∗^	-6.54	29.38 (6.23)	<0.001^∗^	-7.67
	Postinterv.	1.60 (0.67)			34.56 (8.32)			40.11 (9.24)		
Control	Preinterv.	1.28 (0.58)	0.25	1.19	27.29 (7.20)	0.62	-0.50	30.28 (6.88)	0.22	-1.27
	Postinterv.	1.15 (0.63)			27.77 (5.54)			31.18 (6.30)		

SD: standard deviation; Nm: Newton meter; interv.: intervention; *t*: *t* value for the *t*-test (contrast). ^∗^ *P* ≤ 0.05.

## Data Availability

The data of this study is available on request. Requests should be sent to Thales R. Souza at the email address thalesrs@ufmg.br.

## Supplementary Materials

Supplementary Materials Figure S1: measurement of foot-ankle complex varus alignment (forefoot-shank angle): (a) posterior view and (b) lateral view [15]. Table S1: descriptive data (mean and standard deviation) of the kinematic and isokinetic variables before and after the intervention for the intervention and control groups and for the subgroups related to the foot-ankle complex varus alignment.
