# Supplementary material for: Pelvic Drop Changes due to Proximal Muscle Strengthening Depend on Foot-Ankle Varus Alignment
Source: Appl Bionics Biomech. 2019 May 12;2019:2018059. doi: 10.1155/2019/2018059 (PMC6541954; doi:10.1155/2019/2018059)
Supplement: Supplementary Materials — Figure S1: measurement of foot-ankle complex varus alignment (forefoot-shank angle): (a) posterior view and (b) lateral view [15]. Table S1: descriptive data (mean and standard deviation) of the kinematic and isokinetic variables before and after the intervention for the intervention and control groups and for the subgroups related to the foot-ankle complex varus alignment. [file 2018059.f1.pdf]

Supplementary Material

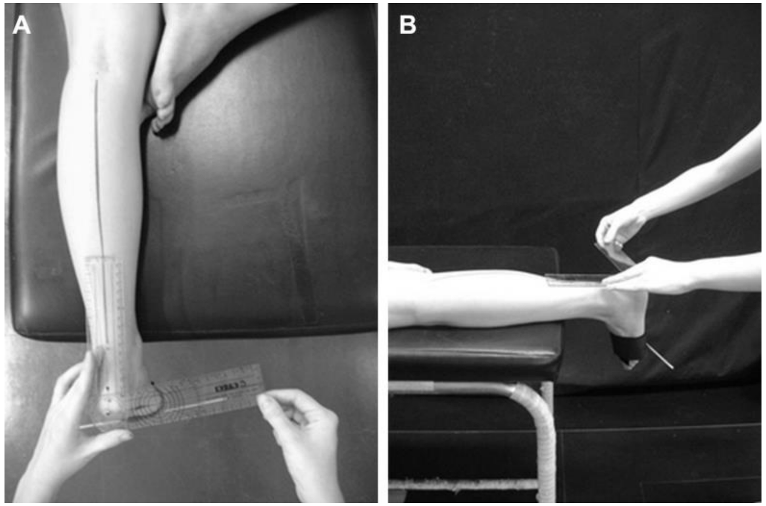

Figure S1- Measurement of foot-ankle complex varus alignment (Forefoot-shank angle):
a) Posterior view b) Lateral view.
Fonte: SOUZA *et al.*, 2014a.

Table S1- Descriptive data of outcome variables

Group and Subgroups	Condition	Passive mean torque (Nm)<br>Mean (SD)	Concentric peak torque (Nm)<br>Mean (SD)	Eccentric peak torque (Nm)<br>Mean (SD)	Pelvic drop excursion (°)<br>Mean (SD)	Pelvic anterior rotation (°)<br>Mean (SD)	Hip adduction(°)<br>Mean (SD)	Hip internal rotation (°)<br>Mean (SD)
**Intervention**	Pre-intervention	1.28 (0.59) *	25.16 (5.03) *	29.38 (6.23)*	5.93 (2.02)	4.39 (2.44)	7.80 (2.63)	4.06 (2.58)
	Post-intervention	1.60 (0.67) *	34.56 (8.32) *	40.11 (9.24)*	5.93 (2.00)	3.71 (2.00)	8.28 (2.42)	4.24 (2.50)
**Control**	Pre-intervention	1.28 (0.58)	27.29 (7.20)	30.28 (6.88)	4.10 (2.02)	4.31 (2.44)	6.41 (2.64)	3.13 (2.58)
	Post-intervention	1.15 (0.63)	27.77 (5.54)	31.18 (6.30)	4.12 (2.00)	3.81 (2.00)	6.78 (2.42)	2.97 (2.51)
**SVI**	Pre-intervention	1.22 (0.50)	25.56 (5.03)	29.79 (6.29)	5.62 (2.11) *	4.66 (3.33)	7.26 (2.50)	3.48 (2.30)
	Post-intervention	1.49 (0.55)	37.24 (4.99)	42.47 (6.99)	5.06 (2.18) *	3.78 (2.65)	7.57 (2.20)	3.71 (2.24)
**LVI**	Pre-intervention	1.35 (0.69)	24.77(5.20)	28.97 (6.39)	6.24 (2.45) ɫ	4.12 (2.21)	8.35 (3.10)	4.64 (3.76)
	Post-intervention	1.72 (0.78)	31.88 (10.19)	37.76 (10.82)	6.80 (2.17) ɫ	3.63 (1.71)	8.99 (2.57)	4.78 (2.94)
**SVC**	Pre-intervention	1.36 (0.68)	29.18 (5.70)	32.48 (5.95)	4.95 (1.42)	3.48 (1.82)	6.96 (2.64)	3.88 (1.62)
	Post-intervention	1.19 (0.66)	29.78 (5.80)	32.78 (6.06)	5.04 (1.42)	3.18 (1.74)	7.60 (2.65)	3.45 (2.50)
**LVC**	Pre-intervention	1.19 (0.48)	25.25 (8.26)	27.92 (7.24)	3.25 (2.03)	5.14 (2.17)	5.86 (2.22)	2.39 (2.21)
	Post-intervention	1.12 (0.61)	25.59 (4.49)	29.45 (6.32)	3.20 (2.16)	4.43 (1.77)	5.96 (2.20)	2.48 (2.28)

LVI: larger varus intervention and SVI: smaller varus intervention; LVC: larger varus control and SVC: smaller varus control; Nm: Newton-meter; (°): degrees.
(*) $P \leq 0.05$; (ɫ) *P* marginal
